# Manual physical therapy and perturbation exercises in knee osteoarthritis

**DOI:** 10.1179/2042618613Y.0000000039

**Published:** 2013-11

**Authors:** Daniel Rhon, Gail Deyle, Norman Gill, Daniel Rendeiro

**Affiliations:** 1 Madigan Army Medical Center, Department of Physical Medicine, Tacoma, WA, USA; 2 Brooke Army Medical Center, San Antonio, TX, USA; 3 Occupational and Physical Therapy Service, Warrior Transition Brigade, Fort Hood, TX, USA

**Keywords:** Knee osteoarthritis, Manual therapy, Perturbation exercises, Physical therapy

## Abstract

**Objectives:**

Knee osteoarthritis (OA) causes disability among the elderly and is often associated with impaired balance and proprioception. Perturbation exercises may help improve these impairments. Although manual physical therapy is generally a well-tolerated treatment for knee OA, perturbation exercises have not been evaluated when used with a manual physical therapy approach. The purpose of this study was to observe tolerance to perturbation exercises and the effect of a manual physical therapy approach with perturbation exercises on patients with knee OA.

**Methods:**

This was a prospective observational cohort study of 15 patients with knee OA. The Western Ontario and McMaster Universities Arthritis Index (WOMAC), global rating of change (GROC), and 72-hour post-treatment tolerance were primary outcome measures. Patients received perturbation balance exercises along with a manual physical therapy approach, twice weekly for 4 weeks. Follow-up evaluation was done at 1, 3, and 6 months after beginning the program.

**Results:**

Mean total WOMAC score significantly improved (*P* = 0.001) after the 4-week program (total WOMAC: initial, 105; 4 weeks, 56; 3 months, 54; 6 months, 57). Mean improvements were similar to previously published trials of manual physical therapy without perturbation exercises. The GROC score showed a minimal clinically important difference (MCID)≥+3 in 13 patients (87%) at 4 weeks, 12 patients (80%) at 3 months, and 9 patients (60%) at 6 months. No patients reported exacerbation of symptoms within 72 hours following each treatment session.

**Discussion:**

A manual physical therapy approach that also included perturbation exercises was well tolerated and resulted in improved outcome scores in patients with knee OA.

## Introduction

Exercise interventions are important in the evidence-based treatment of knee osteoarthritis (OA).^1^–^9^ The goals of exercise for knee OA are typically to improve movement, function, and cardiovascular fitness, while reducing pain and body mass index.^4^,^5^ Impairments of balance, joint proprioception, and kinesthesia are also related to knee OA and may persist even after knee replacement surgery.^10^,^11^ These impairments may result in falls and increased cost of management.^12^ Joint laxity and proprioceptive inaccuracy are predictors of poor functional outcomes.^13^ However, the measurement of proprioceptive deficits has been poorly defined in the literature.^14^

There is limited evidence supporting the efficacy of proprioceptive exercise for patients with knee OA.^15^–^19^ There may be no additional benefit of perturbation and agility training exercises when added to an impairment-based exercise program.^19^ Some even advocate that other approaches, such as task-specific exercises, may have more value than some impairment-based exercise approaches.^18^ Although a case report on perturbation exercises for a patient with knee OA suggested a positive outcome,^20^ perturbation exercises may be poorly tolerated.^16^,^21^,^22^ This may be related to the increased joint compression forces that closed-chain exercises are thought to place on the knee joints.^22^ Other studies suggest that repetitive loading can adversely affect the viability of cartilage in the knee. 21,23 Consideration of the irritability of knee OA symptoms with closed-chain exercises has led to several studies looking at methods of exercise that limit weight through the joints, specifically to improve tolerance.^16^,^17^,^24^,^25^ For example, Lin *et al.*^17^ argued that while closed-chain exercises activate more muscle spindle and joint proprioceptors, they can also lead to an increase in pain, swelling, and inflammation if not properly controlled. Based on this rationale, they sought to provide perturbation exercises to patients with knee OA while seated by way of a computer-facilitated proprioception device. In another study, Jan *et al.*^26^ stated that while perturbation training may be valuable, it can increase pain and inflammation when performed in the standing position. They also sought to evaluate perturbation exercise prescription in a seated position. While perturbation exercises may increase joint load in the knee, we were unable to find any studies that compared joint compression forces from perturbation exercise to other forms of exercise. However, consideration of patient tolerance to prescribed exercise appears to be a valid concern, and this may be why some clinicians avoid perturbation exercises in this population.

Another treatment strategy for knee OA is the manual physical therapy approach, which has demonstrated substantial benefits that can last out to 1 year.^27^–^30^ This approach is based on clinical reasoning and includes highly specific passive manual techniques and therapeutic exercises that support and reinforce those techniques (Appendix 1).^31^ In the context of this approach, the integration of perturbation exercises as a multimodal treatment may lead to improved perturbation training tolerance. Manual therapy has been reported to act, in part, by inhibiting and modulating pain,^32^,^33^ or altering the acute inflammation in response to exercise.^34^ This may lead to an increase in exercise tolerance that would otherwise be lacking or diminished without the combination of manual therapy.

This investigation is the first step in a line of research to ultimately evaluate the effect of perturbation exercises on knee OA. It aims to include effects on patient-centered outcome measures, functional tests, and eventually tests of balance and proprioception with the overarching goal of reducing fall risks. The purpose of this study was to evaluate tolerance to and outcomes associated with the addition of proprioceptive exercises to an already established manual physical therapy approach. If this therapy is appropriate for addressing proprioception impairments, and delivery in conjunction with a manual physical therapy approach can be well tolerated,^35^ then this combined intervention could be a focus for future studies.

## Materials and Methods

### Subjects

This study was a repeated-measures, prospective, observational cohort study. Patients were recruited from a convenience sample of consecutive patients evaluated for knee OA at the Physical Therapy Clinic, Brooke Army Medical Center, San Antonio, Texas from January to May 2008. Patients were treated by licensed physical therapists who were training in an APTA-credentialed manual physical therapy fellowship program. All patients were screened and provided informed consent. Inclusion and exclusion criteria are presented in Table 1. The study was approved by the Brooke Army Medical Center Institutional Review Board.

**Table 1:** Inclusion and exclusion criteria for enrollment in the study

Inclusion criteria
1. Meeting ≥1 of the three classification criteria for knee osteoarthritis (OA) as previously described (sensitivity, 89%; specificity, 88%) *^36^,^37^ a. Knee pain for most days of the prior month and i. Crepitus with active motion (and) ii. Morning stiffness in knee ≤30 minutes (and) iii. Age≥38 years b. Knee pain for most days of the prior month and i. Crepitus with active motion (and) ii. Morning stiffness in knee >30 minutes (and) iii. Bony enlargement c. Knee pain for most days of the prior month and i. No crepitus (and) ii. Bony enlargement
2. Eligible for care in a military medical treatment facility
3. Minimum age 38 years
4. Read, write, and speak sufficient English to complete the outcome tools
Exclusion criteria
1. Only periarticular pain or pain referred from another region; no joint pain
2. Injections to the knee within the last 30 days
3. History of knee joint replacement surgery on involved limb
4. Evidence of other systemic rheumatic condition (rheumatic arthropathies such as lupus, rheumatoid arthritis, psoriasis, or gout)
5. Balance deficits from other non-musculoskeletal conditions (such as neurologic impairments, diabetic neuropathy, cerebellar disorders, or Parkinson disease)

* Altman (1991)^37^ and Altman *et al.* (1986)^36^.

### Instrumentation

The Western Ontario and McMaster Universities arthritis index (WOMAC), a self-administered health status instrument, is valid, reliable, and responsive to change in this population. It has satisfactory test-retest reliability for function, and acceptable overall inter-rater reliability.^38^–^40^ The WOMAC has three clinical subscales (pain, stiffness, and physical function), and lower scores are associated with less pain and stiffness, and better function. The minimal clinically important difference (MCID) for the WOMAC is a change of 12%.^41^

The global rating of change (GROC) is a common, feasible, and useful method for assessing outcome measures and overall changes in quality of life from an established baseline point. It is responsive to change, and has been used in clinical trials for knee OA.^19^,^42^,^43^ The GROC has a 15-point scale, with a score of 0 indicating no change, −1 to −7 indicating worsening of symptoms, and +1 to +7 indicating improvement of symptoms. A change of ≥+3 points indicates the MCID related to a patient’s perception of quality of life.^42^

Tolerance to treatment was assessed by asking patients a series of questions related to their signs and symptoms on the subsequent visit. They were asked if their symptoms had gotten significantly worse at five different time points since their last treatment: (i) immediately after treatment, (ii) several hours after treatment, (iii) that evening before going to bed, (iv) the following morning, and (v) from the following morning until the follow-up which was typically 72 hours later. They were told immediately after each treatment to try and remember how they felt, as they would be asked these questions on their next follow-up.

The functional squat test is a provocative test and measure of function, with excellent intra-rater reliability,^44^ that uses pain and range of motion (ROM) to report its score. In the functional squat test, pain was measured with the 11-point numeric pain rating scale (NPRS) and ROM was measured with a gravity inclinometer (Baseline, Fabrication Enterprises Inc, White Plains, NY).^44^ Patients stood with their feet shoulder-width apart and pointed forward. The top edge of the gravity inclinometer was placed just below the tibial tuberosity and set to 0°. The patients bent their knees and lowered their buttocks straight down toward the heels, without bending forward or letting the heels come off the ground. The knee ROM measurement was taken at the greatest angle at which the patient maintained this posture or stopped because of pain. A 2-point change in the NPRS represented a clinically meaningful change.^45^,^46^ No MCID was available for ROM changes in the functional squat test in this population.

The step-up test is valid and reliable for measuring balance in patients post stroke^47^ and has been used to measure balance impairments in patients with knee OA.^47^,^48^ The step-up test may correlate with functional reach (*r* = 0.68), gait velocity (*r* = 0.83), and stride length (*r* = 0.82) in stroke patients.^47^ There is a significant difference in step-up test ability between patients with knee OA and healthy controls.^48^ The step-up test was performed as previously described, with only one trial allowed for each subject after two practice steps.^48^ Patients stood on the symptomatic leg (or the most symptomatic leg when there was bilateral involvement) and maintained balance while placing the opposite foot from the ground onto a 15-cm step and back onto the ground. A full repetition was defined as the full step-up and step-down movement, with the foot placed fully onto the step and fully back onto the ground. The number of repetitions performed within 15 seconds was recorded. If loss of balance occurred, the test was terminated and the assigned score was the number of steps recorded. This did not occur with any of the patients in this study. No MCID has been established for the step-up test.

### Evaluation

The primary dependent variables were 72-hour tolerance to treatment, the WOMAC, and the GROC. The WOMAC was measured at 0 weeks (initial), and then along with the GROC at 4 weeks, 3 months, and 6 months. The secondary dependent variables were the step-up and functional squat tests measured at 4 weeks, in order to assess functional tasks immediately upon completion of treatment. Another investigator who did not treat the subject verified that the WOMAC was complete and placed it in a locked file. The treating therapist was blinded to all outcome variables throughout the treatment of the study. The initial evaluation included a detailed history, review of systems, and physical examination. The history included questions about the duration, severity, location, and distribution of symptoms. The physical examination included functional tests, palpation of bony landmarks, ROM measurement, muscle length tests, and manual assessment of the joints and soft tissues including the knees, hips, lumbar spine, feet, and ankles.

### Intervention

Patients were treated in the physical therapy clinic twice weekly for 4 weeks (total, 8 sessions). The manual physical therapy approach included joint and soft tissue mobilization (Appendix 1 and online supplementary material 1) with stretching, range of motion, and strengthening exercises that reinforced the manual techniques.^35^ These were also prescribed for the home exercise program. Exercises were chosen that addressed common functional limitations and impairments, and were customized to each subject based on impairments identified during the physical examination, as previously described (Appendix 1).^27^,^28^,^49^

In addition to the manual physical therapy approach, perturbation exercises, modified from a case study (Fig. 2),^20^ were performed at each clinical visit (Appendix 2 and online supplementary material 2). Patients were also given the standard home exercise program used in prior manual therapy trials for knee OA,^27^,^28^,^35^ and tailored to impairments found in each patient.^35^ The progression of the perturbation exercises was guided by clinical reasoning, and varied depending on each patient’s presentation, with careful assessment of the severity and persistence of symptoms in response to a very low initial intensity of perturbation exercises. The first few sessions typically included more emphasis on applying manual treatment and teaching reinforcing exercises. The final sessions included more emphasis on the perturbation exercises (Appendix 2 and online supplementary material 2).

**Figure 1:**
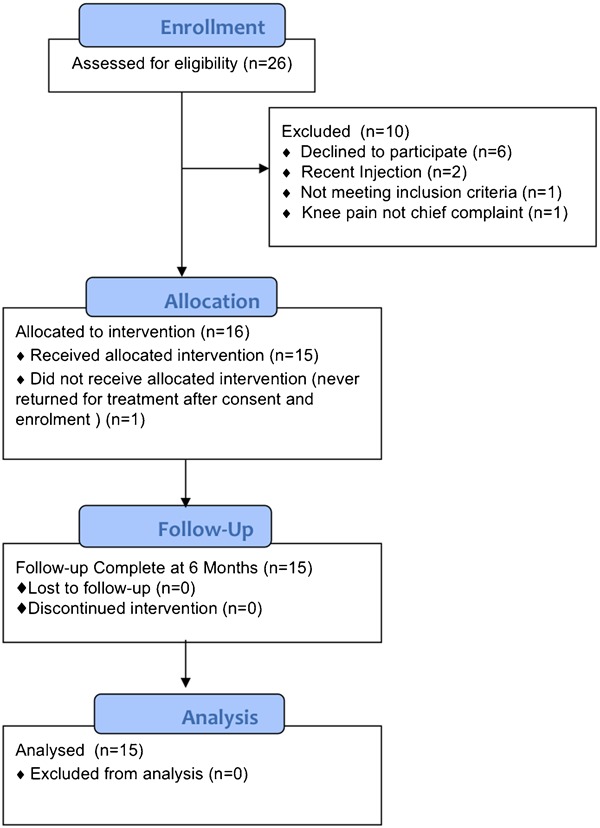
Study flowchart.

**Figure 2:**
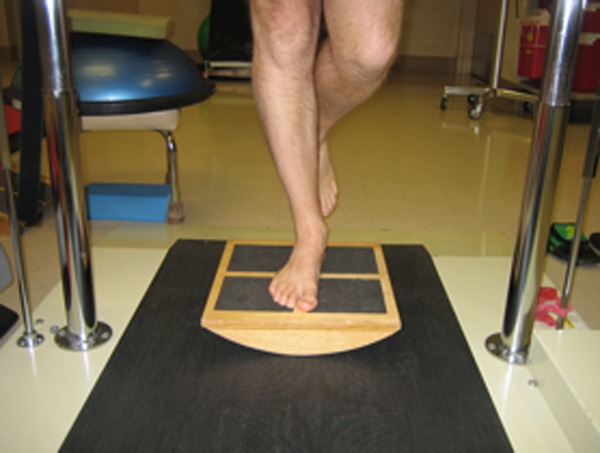
Perturbation challenge exercises.

### Data analysis

Data were analyzed with statistical software (SPSS for Windows 16.0, SPSS Inc., Chicago, IL). Descriptive statistics were calculated on demographic and outcome data. Inferential statistics were calculated for the dependent variables (WOMAC, GROC, functional squat test, and step-up test). The 72-hour response to treatment was calculated descriptively. The independent variable was time. Analysis of variance (ANOVA) was performed for the WOMAC total score at initial time, 4 weeks, 3 months, and 6 months. Separate ANOVA tests were also performed for the WOMAC subscales of pain, stiffness, and function. The Greenhouse–Geisser correction factor was applied when assumptions of sphericity were not accomplished. Post hoc analyses were performed using the least significant difference test for comparisons between different times. The GROC was assessed at 4 weeks, 3 months, and 6 months, and reported as frequency counts of scores achieving no change (≤2 points), clinically important change (≥3 points), and dramatic change (≥6 points). Paired *t* tests were performed for the functional squat test (NPRS and ROM) and step-up test (initial to 4 weeks). Statistical significance was defined by *P*≤0.05.

## Results

During the 3-month period, 26 patients were referred for knee OA. All 16 patients enrolled in the study (Fig. 1) had radiographic signs of knee OA, and 10 had bilateral knee symptoms (Table 2). Visible bony enlargement of the knee joint was noted on clinical observation in 10 patients. Mean total WOMAC score improved significantly, with 46% improvement from initial to 6 months (Table 3). The total WOMAC score was significantly improved at the end of the 4-week treatment (*P* = 0.001), and this improvement remained for 6 months (*P* = 0.009). For all three WOMAC subscales, significant differences from baseline were found at all time points except at the 6 month follow-up for stiffness (Table 3).

**Table 2:** Clinical and demographic features of patients*

	Men	Women	Total
Number of patients	7	8	15
Age (years)	52	57	55
Active duty soldier (*n*)	3	1	4
Duration of symptoms (months)	98	31	60
Height (m)	1.75	1.69	1.72
Body weight (kg)	99	218	218
Body mass index (kg/m^2^)	32	35	34
Body surface area (m^2^)	2.18	2.15	2.16
Most symptomatic knee			
Left	4	4	8
Right	3	4	7
Bilateral involvement	5	5	10
Crepitus present	5	8	13
Morning stiffness			
None	3	0	3
<30 minutes	3	2	5
≥30 minutes	1	6	7
Imaging findings			
Radiographic signs	7	8	15
MRI done	4	1	5
Meniscus abnormal (MRI)	4	1	5
Compartment involvement			
Lateral	3	6	9
Medial	7	6	13
Patellofemoral	4	7	11
Co-morbidities†			
1	7	8	15
≥2	6	4	10
Diabetes mellitus	1	1	2

* *N* = 15 patients. Data reported as mean or number.

† Co-morbidities included additional body regions with marked pain (low back, hip, ankle, neck, or shoulder).

**Table 3:** Outcome measures for patients*

Outcome measures	Initial	4 weeks†	*P*≤‡	3 months	*P*≤‡	6 months	*P*≤‡
Functional squat							
Numeric pain rating scale (NPRS)	5±2	3±2	0.000				
ROM	29±9	35±10	0.001				
Step-up test	9±3	14±4	0.02				
WOMAC							
Stiffness	10 (6.8–12.9)	6 (3.1–8.5)	0.002	5 (2.4–8.4)	0.001	7 (3.3–10.1)	0.083
Pain	22 (16.8–26.2)	10 (4.7–15.0)	0.000	11 (4.3–16.9)	0.004	12 (5.6– 17.4)	0.006
Function	74 (52.5–94.5)	40 (21.7–59.0)	0.001	38 (16.7–58.6)	0.003	39 (17.0–60.8)	0.009
Total (MCID = 12)	105 (77.0–132.7)	56 (30.3–81.7)	0.001	54 (23.7–83.6)	0.003	57 (26.3–87.9)	0.009
GROC							
MCID≥+3		13 (87%)		12 (80%)		9 (60%)	
MCID+6 or +7		7 (47%)		7 (47%)		7 (47%)	

* Reported as mean±SD; mean (95% confidence interval); or number (%). Abbreviations: GROC, global rating of change; MCID, minimal clinically important difference; ROM, range of motion in degrees; WOMAC, Western Ontario and McMaster Universities osteoarthritis index.

† Functional tests performed only initially and at 4 weeks.

‡ Comparison against initial value.

The GROC score showed marked improvement with 87% of the patients reporting a clinically important improvement (GROC≥+3) at the 1-month follow-up, 80% at the 3-month follow-up, and 60% at the 6-month follow-up point. Nearly half (47%) reporting dramatic change (GROC≥+6) at all time points (Table 3). The two functional tests were only assessed immediately after the treatment regimen and compared to baseline. The functional squat test had significant improvement in both mean NPRS and ROM from initial to 4 weeks (Table 3). The mean step-up test improved significantly from initial to 4 weeks, with a mean improvement of 4–5 steps during the 15 second test (Table 3). All 15 patients who received treatment were compliant with all follow-up appointments during the study.

## Discussion

In the present series of patients with knee OA, a manual physical therapy approach incorporating perturbation exercises resulted in significant improvement in all outcome scores and functional tests. The mean 46% improvement in total WOMAC score from initial to 6 months is well above the MCID of 12% and is consistent with previous trials using the same manual therapy approach without perturbation exercises.^27^,^28^ Improvements in the GROC score, step-up test, and functional squat test also were significant. These results suggest that the addition of carefully applied perturbation exercises within the context of a manual therapy approach may be well tolerated and a reasonable treatment delivery strategy. These results lay groundwork for future research to directly compare a manual therapy approach with and without perturbation exercises, a manual therapy approach with perturbation exercises to a functional exercise approach with perturbation exercises, and to investigate other outcome measures that appropriately measure balance, proprioception, stumble response, and ultimately falls.

By 6 months five patients had received knee joint injections of either corticosteroid or viscosupplementation and two of those same patients received arthroscopic surgery. Arthroscopic surgery was done during the study in two patients (one patient with a more symptomatic knee, and one with a less symptomatic knee initially). Pain medication was used by 12 patients initially (10 patients daily; 2 patients as needed), including non-steroidal anti-inflammatory drugs and/or acetaminophen. At each of the follow-up points fewer patients were taking medications than at baseline (4 weeks and 3 months, 7; 6 months, 10). There were no adverse events or reports of acute flare-ups during treatment or within 72 hours after each treatment in any subject.

The risk of falls in patients with knee OA^12^,^50^–^52^ has been attributed, in part, to decreased balance, agility, muscle function, proprioception, and the ability to respond to perturbations.^10^,^14^,^53^–^57^ Therefore, it may be important to design interventions to address these impairments, with careful attention to the type and dose of exercise to address balance and proprioception.^14^,^58^,^59^ Manual physical therapy as an effective treatment approach for knee OA has been well established.^27^–^30^ It has been shown to improve pain and function for at least 1 year, in multiple settings, and in patients with or without concurrent meniscus tears.^27^–^30^ Perturbation and agility training may improve proprioceptive deficits, but it is unknown whether addressing balance and proprioceptive deficits will actually decrease the risk of falls. While more research is needed to determine this, our study is the first in this line of research demonstrating that an intensive perturbation training program may be undertaken, within the context of a manual physical therapy approach, without apparent irritation or increase in pain or disturbance of functional outcomes.

Substantial improvement in the pain and function subscales of the WOMAC, along with no report of increased joint irritation in the 72 hours following each treatment, suggest that the exercises were well tolerated and not associated with adverse effects. As increased joint inflammation and effusion may decrease proprioception, it is important that all aspects of a knee OA treatment program be well tolerated.^53^ The observations from the present study suggest that perturbation exercises in the weight bearing position can be safely added to a manual physical therapy approach, using clinical reasoning to adjust individually for dose and progression, in patients with knee OA.

There is no solid consensus on the exact mechanisms resulting from manual physical therapy that result in therapeutic benefits. However, it is likely that it works through both biomechanical and neurophysiological mechanisms.^60^ The clinical trials that demonstrated the effectiveness of manual therapy for improving pain and function in patients with knee OA did not speculate on specific potential mechanisms other than suggesting that the effects of manual therapy may be derived from treating the spectrum of tissues in and around the knee and other related body regions.^27^,^28^ The knee has proprioceptive mechanoreceptors that may be damaged from the degenerating joint process common in OA.^61^,^62^ Dysfunction within these neural structures may mediate weakness and instability in joints affected by OA and negatively affect proprioception.^63^ Manual physical therapy has also been reported to inhibit and modulate pain,^32^,^33^ induce a controlled inflammatory response that initiates healing and influences processing of pain,^64^–^66^ and alter acute inflammation in response to exercise.^34^ These could all contribute to decreased pain from muscle contraction, improving tolerance for exercise. Joint mobilizations also may modulate proprioceptive input to joint structures, prime the joint and surrounding muscles for optimal response to strengthening programs, and improve muscle control and reaction times.^67^,^68^ These are all possible mechanisms contributing to the improvements seen with the patients in this cohort. However, we do not know if perturbation training is tolerated better when prescribed in conjunction with manual therapy, or the additional effect of this multimodal treatment on balance and functional measures of proprioception. This may be an important area to consider in future research related to perturbation training.

Limitations of the present study include a cohort study design with no comparison group, therefore no cause-and-effect relationship can be assessed. In addition, five patients received viscosupplementation or corticosteroid injections to the knee, and two of those also had arthroscopic surgery during the 6-month follow-up period. While this may confound the results, only three of these additional procedures (injections) occurred during the initial 1-month period of treatment, and two of these patients had no improvement in their WOMAC scores at the 4-week follow-up. Both of the arthroscopic surgeries occurred at the 3-month mark. All of the patients responded that they felt no significant change in symptoms after their injection or arthroscopic surgery procedure. Also, four of the five patients stated that these procedures had already been considered as part of their treatment management plan before they were referred to physical therapy. However, they did not make this known until the end of the study when asked about the reasons for pursuing surgery when they seemed to be improving with the physical therapy program. While we may not fully understand what drives these patient behaviors, this is not isolated to our study alone. In a recent randomized trial comparing physical therapy to surgery, 30% of subjects randomized to receive physical therapy crossed over to the surgery group, despite mean improvement in the physical therapy group being equal to that of the surgery group.^30^ Therefore, these decisions may not have been made due to a lack of improvement with the manual therapy and exercise program. This may be a separate focus for future research. In addition, it is unknown whether the present intervention improved impairments in proprioception and balance, which were assessed only indirectly with the step-up test.

In summary, a manual physical therapy approach including perturbation exercises in a symptomatic knee OA cohort was well tolerated. It was also associated with improved pain, function, and balance as previously noted with manual physical therapy alone. This is an important first step in describing a combined intervention, which can be studied within the context of future clinical trials to determine efficacy related to pain, function, balance, and falls compared to other physical therapy or medical approaches.

## Conflict of Interest

None of the authors have any disclosures to make regarding any actual or perceived conflict of interest related to this research report.

## Disclaimer

The views expressed are those of the authors and do not reflect the official policy of the Department of the Army, the Department of Defense, or the United States Government.

## Appendix 1: Manual physical therapy program

The manual physical therapy program included a passive manual examination, followed by tailored manual treatment techniques, and then reinforced with supporting exercises.^31^ To begin, a passive manual examination was performed on each knee. Joints were progressively stressed to demonstrate impaired movement or to reproduce symptoms comparable to the patient’s primary pain complaint. Maitland grading system^69^ was used to clear the joints in single and combined motion planes; grade IV– indicates the point in the range of movement where resistance to motion begins, and grade IV++ indicates the end-range resistance of the joint. A joint was considered cleared if movement was normal, no pain could be identified throughout the ROM, and if the joint could be taken to a grade IV++ (end-range resistance of joint) without reproducing the subject’s symptoms. If the tibiofemoral joint for example, could be cleared in one plane (isolated plane of flexion or extension), then the therapist attempted to clear the joint in a combined plane. This was performed by adding a combined movement such as a varus force with tibial adduction or a valgus force with tibial abduction to the end-range of flexion or extension. This detailed movement and symptom examination helped identify impairments in any aspect of the knee and ensure thorough assessment before declaring a joint clear.

Any joint movements that were not cleared were documented and formed the basis for choosing the mobilization techniques and dosage that each subject would receive for an intervention. Over the course of several treatment sessions a joint that was not initially cleared could become cleared when impaired movements or symptoms were no longer reproduced with a grade IV++ (end-range) mobilization. Remaining treatment session would then focus on the residual impairments to movement and the symptoms of the patient. If symptoms that were reproduced in the first or second treatment session improved after several treatments, the treating physical therapist progressed the manual intervention to combinations of accessory and physiological movements as described earlier.

Reinforcement exercises were given based on the impairments identified. When patients presented with restriction of knee extension or flexion, terminal knee extension or flexion ROM exercises were taught to reinforce the knee mobilizations. Hip flexor, quadriceps, hamstring, and calf muscle length tightness were common impairments in these patients, and these were addressed with manual stretching techniques and self-stretching exercises. The patellofemoral and proximal tibiofibular joints were also manually assessed for stiffness and symptom reproduction. Mobilizations to these joints were targeted to impairments found on examination, and included a progression of medial, lateral, superior, inferior, or rotatory glides of the patella and anterior-to-posterior and posterior-to-anterior glides of the proximal tibiofibular joint.

Manual physical therapy – video demonstration found in online supplementary material 1:

1. Knee extension mobilizations, grade IV in single plane. Combined movements into varus/abduction or valgus/adduction were added as a progression. This was performed for joint motion evaluation and treatment.
2. Knee extension mobilizations, grade III in single plane. Combined movements into varus/abduction or valgus/adduction were added as a progression. This was performed for joint motion evaluation and treatment.
3. Knee flexion mobilizations, grade IV in single plane. Combined movements into varus/abduction or valgus/adduction were added as a progression. This was performed for joint motion evaluation and treatment.
4. Knee flexion mobilizations, grade III in single plane. Combined movements into varus/abduction or valgus/adduction were added as a progression. This was performed for joint motion evaluation and treatment.
5. Knee flexion mobilizations, grade III with popliteal wedge modification.
6. Patellar mobilizations: medial-lateral glide, medial-lateral rotation, and inferior glide with distraction.

## Appendix 2: Perturbation exercise progression

Patients removed their shoes and stood without any equipment. They received unpredictable perturbation exercises in medial, lateral, anterior, and posterior directions. The patients placed their arms out in front, parallel to the ground over the therapist’s shoulders, without touching the therapist; this would enable them to support themselves when they lost balance. The therapist was positioned to stabilize the subject when the subject began to lose balance. If the initial movement was tolerated, the patient progressed to single-limb stance. The subject progressed to standing on the 2-inch foam, the wooden rocker board, and the foam that was placed on top of the rocker board. The stance was progressed from double- to single-limb stance. Assessment of symptoms was ongoing to minimize flare-ups during the perturbation training. Careful questioning at each session helped to determine if the previous session was well tolerated or if latent pain occurred despite the careful assessment during treatment.

Perturbation exercise – video demonstration found in online supplementary material 2:

Demonstration of balance challenge and perturbation exercise progression.
